# Supplementary material for: Integrative Structural Modeling of Intrinsically Disordered Regions in a Human HDAC2 Chromatin Remodeling Complex
Source: bioRxiv. 2026 Mar 25:2025.08.08.669391. Preprint. [Version 2] doi: 10.1101/2025.08.08.669391 (PMC13041959; doi:10.1101/2025.08.08.669391)
Supplement: Supplement 8 [file NIHPP2025.08.08.669391v2-supplement-8.pdf]

bioRxiv preprint doi: https://doi.org/10.1101/2025.08.08.669391; this version posted March 25, 2026. The copyright holder for this preprint (which was not certified by peer review) is the author/funder, who has granted bioRxiv a license to display the preprint in perpetuity. It is made available under aCC-BY 4.0 International license.

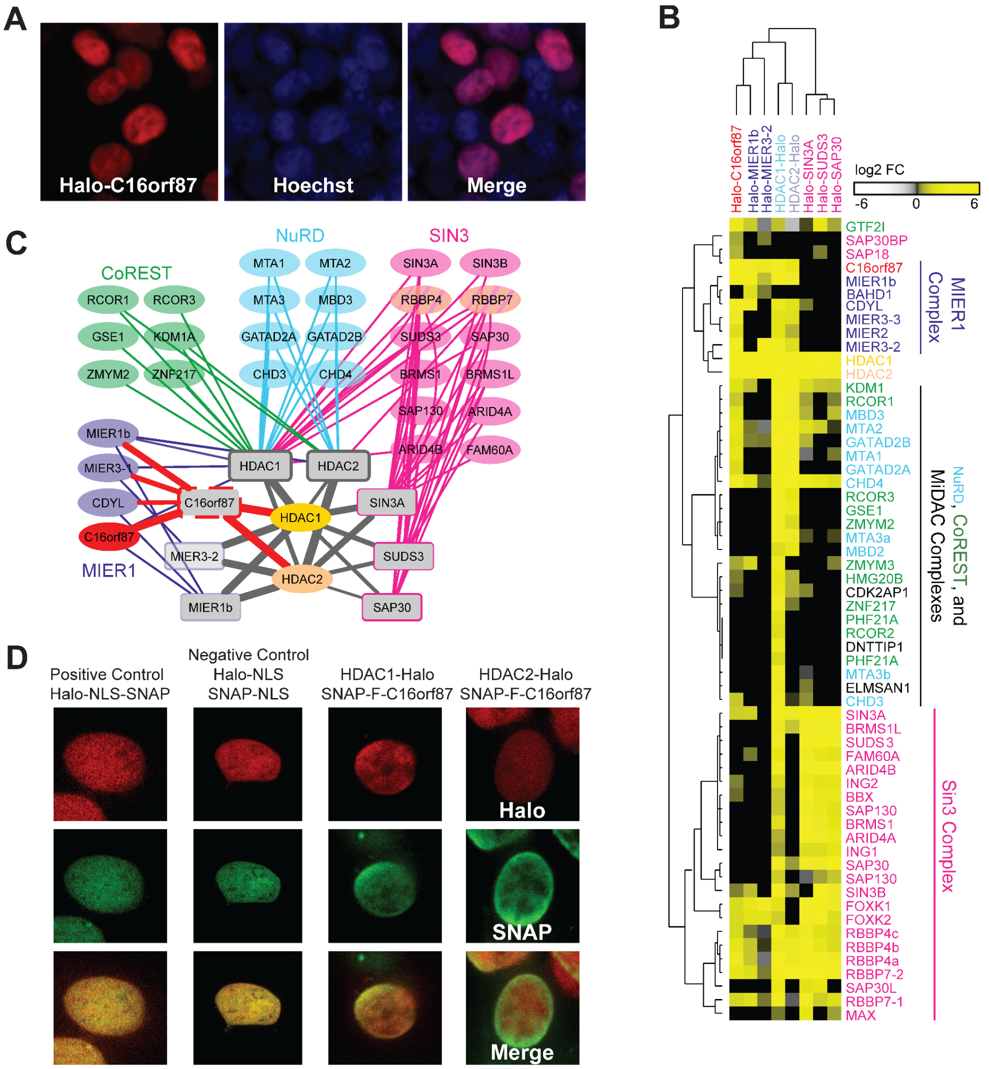

Supplemental Figure S1. C16orf87 localization and interaction network. **A**. Nuclear Localization of HALO-C16orf87 in HEK293T. Halo-tagged proteins are labeled with HaloTag® TMR ligand (red). Nuclei are stained with Hoechst dye (blue). **B**. Heatmap plotting relative protein abundance expressed as log2FC with the hierarchical bi-clustering of the 8 Halo-tagged baits: C16orf87, MIER1b, MIER1-2, HDAC1, HDAC2, SIN3A, SUDS3, SAP30, with the brightest yellow indicating high log2FC (Suppl. Table S1A). **C**. A network analysis was performed using Cytoscape on members of HDAC1/2 associated complexes significantly enriched with at least one of the bait proteins compared to controls (log2FC > 3, FDRup< 0.05; Suppl. Table S1A). The Halo-tagged bait proteins (C16orf87, MIER1b, MIER1-2, HDAC1, HDAC2, SIN3A, SUDS3, and SAP30) are source nodes in gray, with SIN3A associated proteins in pink, NuRD associated proteins in light blue, CoREST associated proteins in green, MIER associated proteins in purple, HDAC1/2 (in light yellow/orange, and C16orf87 in red. **D**. Representative images of acceptor and donor fluorescence for each sample in the AP-FRET experiments. SNAP-F-C16orf87 is labeled with the SNAP-Cell® 505-Star ligand, while HDAC1/2-Halo are labeled with the HaloTag® MR ligand (see data in Suppl. Table S2A).

bioRxiv preprint doi: https://doi.org/10.1101/2025.08.08.669391; this version posted March 25, 2026. The copyright holder for this preprint (which was not certified by peer review) is the author/funder, who has granted bioRxiv a license to display the preprint in perpetuity. It is made available under aCC-BY 4.0 International license.

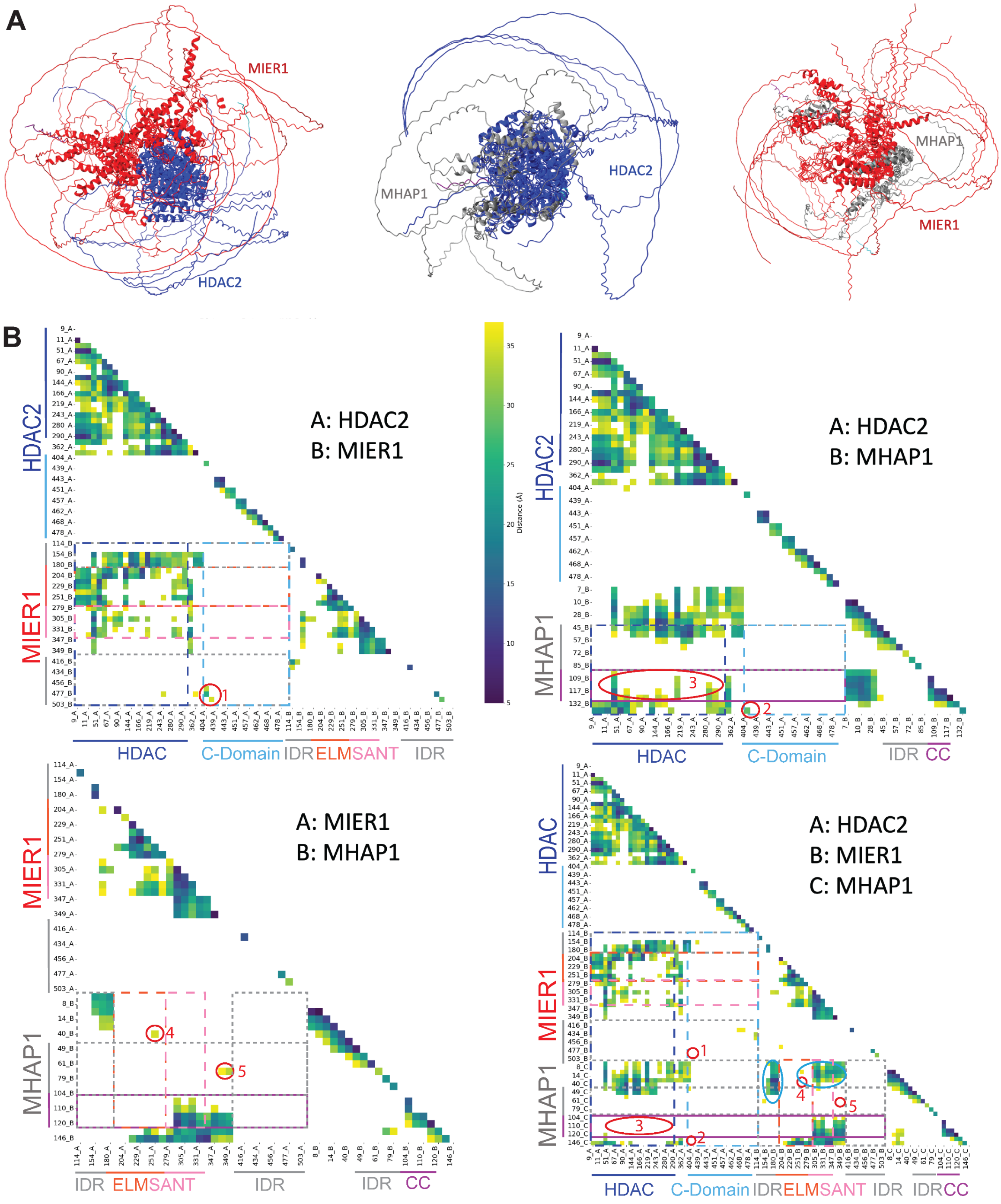

Supplemental Figure S2. Structural Comparison of Alpha-Fold-Based Dimer and Trimer Models. A. Five AF-predicted assemblies are superimposed for each of the dimeric models. **B.** Lysine-lysine interactions within 30Å are plotted for each model. Known structural or IDR domains are indicated by colored lines along the axes. Numbered areas of difference between the dimers and trimer are circled in red and blue.

bioRxiv preprint doi: https://doi.org/10.1101/2025.08.08.669391; this version posted March 25, 2026. The copyright holder for this preprint (which was not certified by peer review) is the author/funder, who has granted bioRxiv a license to display the preprint in perpetuity. It is made available under aCC-BY 4.0 International license.

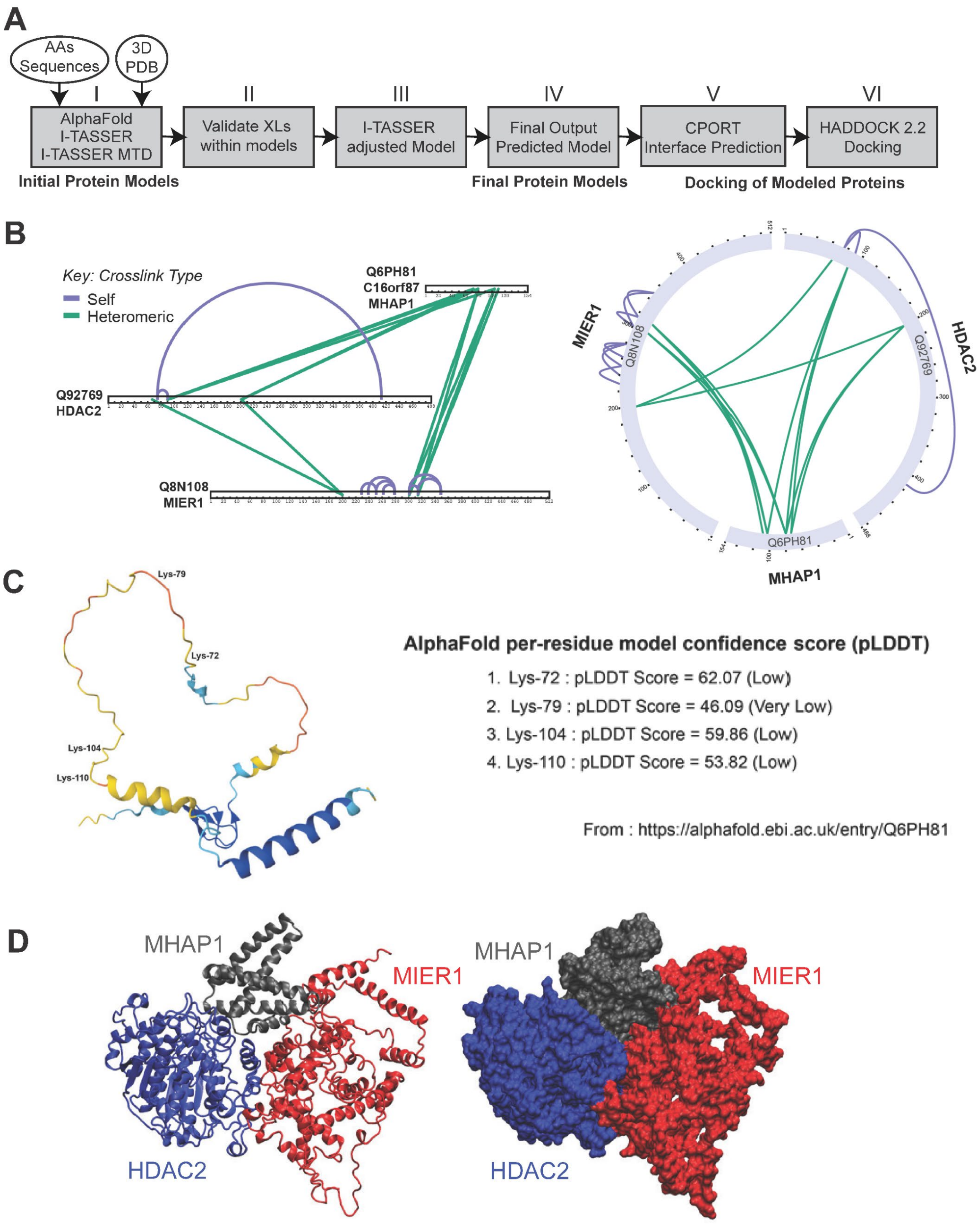

Supplemental Figure S3. XLMS-Guided Integrative Structural Modeling of the MHAP1:MIER1:HDAC2 Trimer. A. *de novo* protein prediction and docking workflow implemented to model the 3D structures of individual proteins and their interaction interfaces and docking in the final complex. **B.** Linear and Circos plots of the crosslinks observed in the XLMS analysis of MHAP1-Halo purifications (see Supp. Table S3). **C.** The four lysine residues found crosslinked in UPF0547 protein C16orf87 in this study are listed with their AlphaFold per-residues scores (from AF-Q6PH81-F1-v4). **D.** Trimeric assembly predicted after step VI of the ISM workflow for HDAC1, MIER1, and MHAP1 (visualized in Chimera as ribbon and space-filled representations).

bioRxiv preprint doi: https://doi.org/10.1101/2025.08.08.669391; this version posted March 25, 2026. The copyright holder for this preprint (which was not certified by peer review) is the author/funder, who has granted bioRxiv a license to display the preprint in perpetuity. It is made available under aCC-BY 4.0 International license.

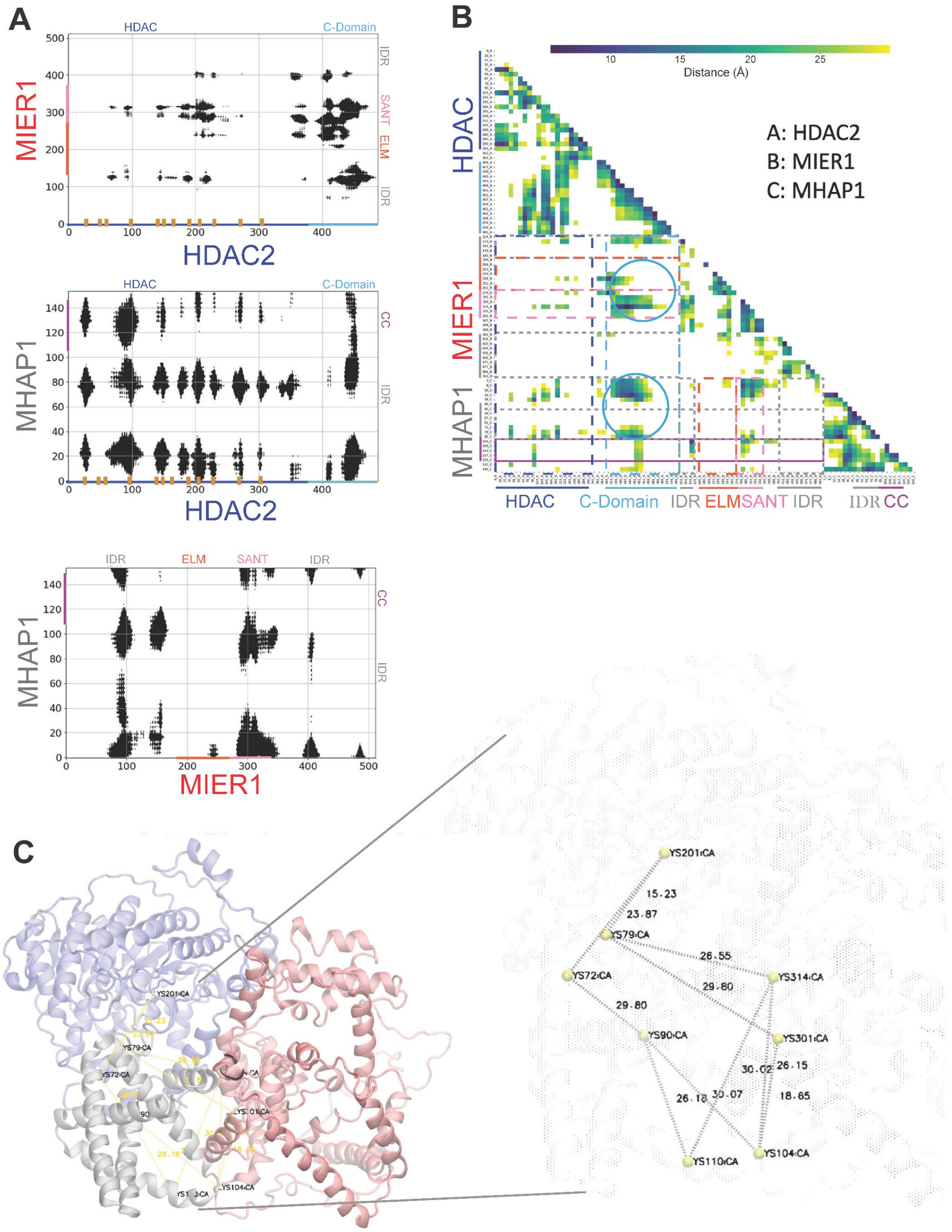

Supplemental Figure S4. Contact Maps and Core Distances within the ISM-Trimer

A-B. Binary residue contact maps (with HDAC ligand binding sites shown as brown tick marks along the x-axis) and heatmap of lysine-lysine distances (within 30 Å) with key interacting hotspots between HDAC C-domain and MIER1 and MHAP1 circled in blue. **C.** Euclidian distances between the α-carbons of the lysine residues (circled in **B**.) forming the stable core of the ISM-predicted ternary assembly, with a scaled-up area of the distances measurements shown on the right.

bioRxiv preprint doi: https://doi.org/10.1101/2025.08.08.669391; this version posted March 25, 2026. The copyright holder for this preprint (which was not certified by peer review) is the author/funder, who has granted bioRxiv a license to display the preprint in perpetuity. It is made available under aCC-BY 4.0 International license.

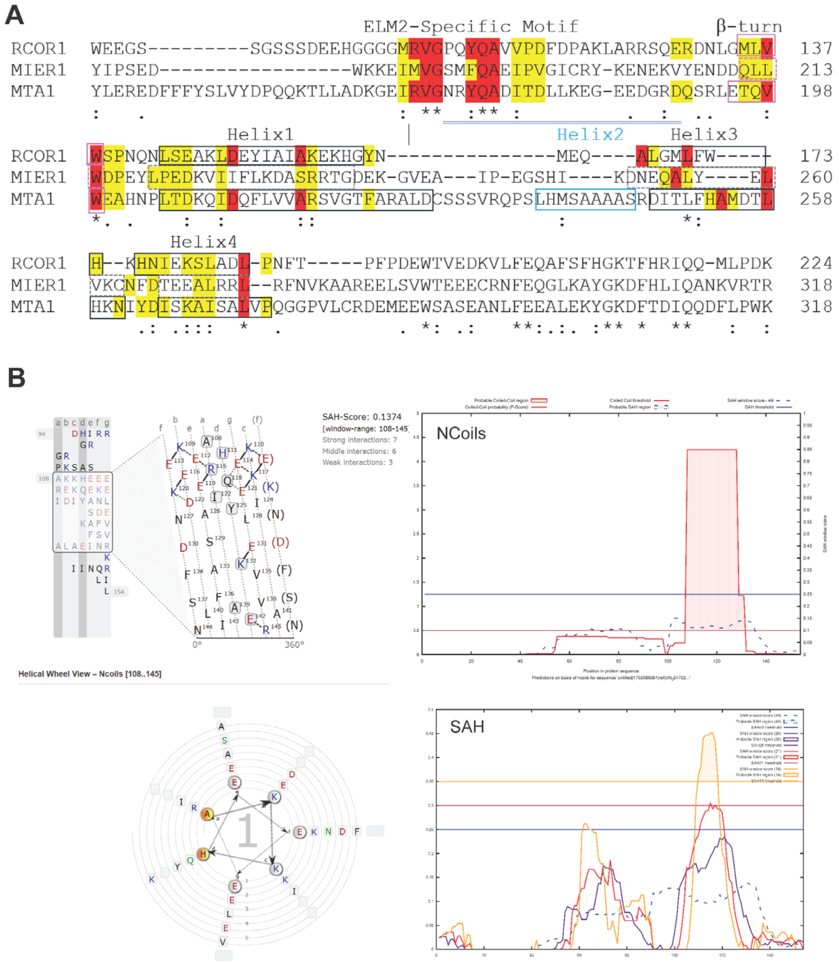

Supplemental Figure S5. Secondary Structures Analyses of MIER1 ELM2 and MHAP1 CC Domains

A. Clustal Omega sequence alignment of ELM2 domains in the RCOR1–3, MIER1, and MTA1–3 proteins. Identical residues are shown in red and conserved residues are shown in yellow. The predicted secondary structures of RCOR1 and the secondary structures of MTA1 are indicated as boxed amino acids, with helix numbers corresponding to the solved MTA1 ELM2 domain. The predicted secondary structures for MIER1 are dashed. **B.** N-Coils helical net and wheel views and prediction results from the MHAP1 sequence to distinguish coiled-coil (CC) from stable a-helix (SAH) domains, all obtained from the Waggawagga prediction algorithm (https://waggawagga.motorprotein.de/).
